# First evidence of anticancer and antimicrobial activity in Mediterranean mesopelagic species

**DOI:** 10.1038/s41598-020-61515-z

**Published:** 2020-03-18

**Authors:** Chiara Lauritano, Kevin A. Martínez, Pietro Battaglia, Antonia Granata, Mercedes de la Cruz, Bastien Cautain, Jesús Martín, Fernando Reyes, Adrianna Ianora, Letterio Guglielmo

**Affiliations:** 1 Marine Biotechnology Department, Stazione Zoologica Anton Dohrn, Naples, Italy; 2 Integrative Marine Ecology Department, Stazione Zoologica Anton Dohrn, Messina, Italy; 3 Department of Chemical, Biological, Pharmaceutical and Environmental Sciences (ChiBioFarAm), University of Messina, Messina, Italy; 4 Fundación MEDINA, Centro de Excelencia en Investigación de Medicamentos Innovadores en Andalucía, Avda. del Conocimiento 34, Granada, 18016 Spain

**Keywords:** Biological techniques, Drug discovery, Ecology

## Abstract

Mesopelagic organisms form huge biomass aggregations, supporting important pelagic trophic webs and several top predators. Although some studies on the occurrence, biology and ecology of these organisms are available, to date there are no investigations on their potential use for anticancer and antimicrobial biotechnological applications. The aim of this study was to screen extracts of seven mesopelagic species for possible anticancer (Lung cell line A549, skin cell line A2058, liver cell line HepG2, breast cell line MCF7 and pancreas cell line MiaPaca-2) and antibacterial (Gram-negative bacteria *Escherichia coli* and *Klebsiella pneumoniae*, the Gram-positive bacteria methicillin resistant/sensitive *Staphylococcus aureus*, and *Mycobacterium tuberculosis*) activities. Results showed that only two species were active, the lanternfish *Myctophum punctatum* and the Mediterranean krill *Meganyctiphanes norvegica*. In particular, *M. punctatum* showed strong activity against the A549 and MCF7 cells, while *M. norvegica* was more active against HepG2 cells. Regarding antibacterial assays, both species were active against methicillin resistant *S. aureus*. Fractionation and LC/MS dereplication of the fractions showed that the main compounds found in extracts of both species were EPA, DHA and ETA. For some of the detected compounds anticancer and/or antibacterial activity are already known, but this is the first time that such activities have been found for mesopelagic species.

## Introduction

The mesopelagic zone, from “meso” meaning intermediate, represents a water layer of the ocean below the epipelagic and above the bathypelagic zones, at depths between 200 and 1000 m, that is characterized by increased hydrostatic pressure, diminished light, high inorganic nutrient concentrations and episodic food supply^1^. A large number of marine organisms live in the mesopelagic zone, from bacteria to zooplankton and nekton, including species adapted to peculiar conditions, such as the twilight environment, inhabited by a myriad of bioluminescent and unique organisms^2–4^. These organisms often form huge biomass aggregations, supporting important pelagic trophic webs and several top predators^5–8^. In particular, due to their high density and wide diffusion, mesopelagic fish can be considered the most abundant vertebrates on earth^9^.

Studies on mesopelagic micronekton have been mainly focused on their distribution, biology and ecology as well as bioluminescent properties but studies related to their potential biotechnological applications are very rare. The reason is mainly due to high costs for their sampling, often requiring the planning of research cruises, the scarce scientific information on most species, and the very scarce available information on their genomes and transcriptomes. Few mesopelagic species are accidentally caught by fishing gear. However, in the Straits of Messina (central Mediterranean Sea), there is a recurrent phenomenon of stranding of mesopelagic organisms due to several factors. Tidal currents, lunar phases, winds and seasons influence the frequency of occurrence of the stranding of mesopelagic species^10,11^.

Fishes are typically both prey and predators, with specialized body shapes, behavioral characteristics and chemical means for defence and “communication” with competing species^12^. The deep-sea environment has been found to be a source of very potent marine-derived agents with bioactive properties, such as Salinosporamide-A and Marizomib, produced by the actinomycete *Salinispora* with promising anticancer activity and currently undergoing Phase III clinical trials against several types of cancer^13,14^. Currently there is only one approved marine drug named Lovaza® from marine fish, whose active principle is composed of omega-3-acid ethyl esters, indicated as an adjunct to diet to reduce triglyceride levels in adult patients with severe hypertriglyceridemia.

However, to our knowledge, mesopelagic species have never been reported to be active for the treatment of human pathologies. Finding new sources of bioactive molecules is of utmost importance, especially for drug-resistant pathologies. The constant appearance and evolution of new antibiotic resistant organisms are the strong motivation for the search for new bioactive compounds from poorly studied environments.

The aim of this study was to investigate extracts of various Mediterranean mesopelagic species for potential anticancer and antimicrobial activities and to perform dereplication of the active extracts in order to describe their most abundant chemical components. Selected samples were mainly from naturally stranded species in the Straits of Messina. This strategy followed an eco-friendly approach that “re-cycles” natural fish waste in order to obtain useful goods. In particular, 7 mesopelagic species were screened for possible antiproliferative activities against a panel of 5 different human cancer cell lines: human lung carcinoma A549, human skin melanoma A2058, hepatocyte carcinoma HepG2, breast adenocarcinoma MCF7 and pancreas carcinoma MiaPaca-2. In addition, growth inhibition activities against *Escherichia coli*, *Klebsiella pneumoniae*, methicillin resistant *Staphylococcus aureus*, methicillin sensitive *Staphylococcus aureus* and *Mycobacterium tuberculosis* were evaluated as well. These experiments will give a broad overview of antimicrobial and anticancer activities of poorly studied mesopelagic species.

## Materials and Methods

### Sampling

The study included the screening of one mesopelagic crustacean *Meganyctiphanes norvegica* (Family Euphausiidae) and six mesopelagic fishes belonging to different families, i.e. *Hygophum benoiti, Lampanyctus crocodilus* and *Myctophum punctatum* (Family Myctophidae), *Argyropelecus hemigymnus* (Family Sternoptychidae), *Chauliodus sloani* and *Stomias boa* (Family Stomiidae). In particular, *M. norvegica, H. benoiti, M. punctatum, A. hemigymnus* and *C. sloani* were collected among stranded organisms in the upwelling area of the Straits of Messina (central Mediterranean Sea), whereas *Stomias boa* and *Lampanyctus crocodilus* were collected from fishing discard landed by a commercial bottom trawl fishing vessel. Therefore, all specimens were already dead when they were collected and there were no ethical issues. For each species, at least triplicates were sampled, measured, weighted, frozen in liquid nitrogen and then kept at −80 °C until chemical extraction (details on samples are reported in Table 1). Exceptions were *C. sloani* and *L. crocodilus*, for which only one individual (of about 8 and 16 g, respectively) was used. Of these two species there are only technical triplicates and not biological triplicates.

**Table 1:** List of species analysed in this study, with information on the number of examined individuals, their size (total length, TL mm, for Crustacea; standard length, SL mm, for Actinopterygii) and weight (g) ranges.

Taxonomic group/Family	Species name	Number of individuals	Length range (mm)	Weight range (g)
***Crustacea***				
Euphausiidae	*Meganyctiphanes norvegica* (M. Sars, 1857)	3	23.8–27.4	0.21–0.25
***Actinopterygii***				
Myctophidae	*Hygophum benoiti* (Cocco, 1838)	3	44.3–51.5	1.61–2.65
	*Myctophum punctatum* Rafinesque, 1810	3	62.3–68.3	3.0–3.9
	*Lampanyctus crocodilus* (Risso, 1810)	1	120.5	16
Sternoptychidae	*Argyropelecus hemygimnus* Cocco 1829	3	18.4–31.5	0.15–0.78
Stomiidae	*Chauliodus sloani* Bloch & Schneider, 1810	1	145.8	7.8
	*Stomias boa boa* (Risso, 1810)	3	165.1–192.1	8.1–13.5

### Chemical extraction

To prepare chemical extracts, each species was frozen with liquid nitrogen and powdered by using a clean mortar and pestle. Chemical extraction was performed as in D’Ippolito *et al*.^15^. Briefly, 2 ml of distilled water were added to each g of powdered fish sample. The same volume of acetone was added and samples were centrifuged at 3600 rpm for 6′ at 4 °C. The supernatant was transferred into sterile tubes and stored on ice. Water and acetone were added again to the remaining pellets and the centrifugation step was repeated for another 2 times. A volume of dichloromethane was added to the recovered supernatant, mixed and centrifuged at 3600 rpm for 6′ at 15 °C. The extraction with dichloromethane was repeated another 2 times. The obtained extracts were treated with anhydrous sodium sulphate in order to remove residues of water and dried using a rotary evaporator under reduced pressure. Extracts were stored at −20 °C until screening.

### Anticancer assays

Whole organism chemical extracts (in triplicates) were screened against a panel of 5 different cancer cell lines (human lung carcinoma A549 ATCC® CCL-185™, human skin melanoma A2058 ATCC® CRL-11147™, hepatocyte carcinoma HepG2 ATCC® HB-8065™, breast adenocarcinoma MCF7 ATCC® HTB-22™ and pancreas carcinoma MiaPaca-2 ATCC® CRL-1420™). The medium composition was different for each type of cell line (as reported in Audoin *et al*.^16^). A549 cells were grown in Ham’s F12K medium with 2 mM Glutamine, 10% Foetal Bovine Serum (FBS), 100 U/mL penicillin and 100 µg/mL streptomycin. A2058 and HepG2 were grown in ATCC formulated Eagle’s M essential medium (MEM) with 10% FBS, 2 mM L-glutamine, 1 mM sodium pyruvate and 100 µM MEM-non essential aminoacids. MCF-7 cells were grown in the previous medium supplemented with 0.01 mg/mL of bovine insulin. MiaPaca-2 cells were grown in Dulbecco’s Modified Eagle’s Medium (DMEM) with 10% FBS, 100 U/mL penicillin and 100 µg/mL streptomycin. The extracts were freeze-dried at 0–1 psi for 4 hours before testing. Significant amounts of extraction solvents should not remain after this process. The solvent used for the test was DMSO. Extracts were dissolved in 100% DMSO, and further diluted following 10-point serial dilutions (1:1 dilutions) to use them as stock solutions for the bioassays (maximum 0.5% DMSO concentration in the tested samples).

The anticancer activity was assessed as reported in Audoin *et al*.^16^ after 72 h exposure with different concentrations of extracts from mesopelagic organisms. Briefly, the cells were seeded in 96-well microtiter plates at a cell density of 1 × 10^4^ cells/well, and incubated for 24 h at 37 °C, 90% humidity and 5% CO_2_ to allow the cell adhesion in the plates. After 24 h, the medium was removed and cells were treated by adding fresh medium and extracts at 0.78125, 1.5625, 3.125, 6.25, 12.5, 25, 50, 100 µg/mL. Each sample and concentration was tested in triplicate. After 72 h, cell viability was assessed using the MTT test (3-(4,5-dimethyl-2-thizolyl)-2,5-diphenyl-2H-tetrazolium bromide). The medium was replaced with medium containing MTT at 0.5 mg/mL and plates were incubated for 3 h at 37 °C. The supernatant was removed and 100 µL of 100% DMSO were added to each well to dissolve the formazan precipitates for 30 minutes at room temperature. Absorbance was measured at OD = 570 nm with a microplate reader (Perkin Elmer Wallac 1420 VICTOR2™ multilabel plate reader). Cell survival was expressed as a percentage of viable cells in the presence of the tested samples, with respect to untreated control cultures. The standard consists of a dose-response curve using doxorubicin (reference drug) at 5 mM with an 8-point serial dilution (1:2 dilutions). Methyl methanesulfonate (MMS) 8 mM was used as the positive control and DMSO 0.5% (diluted 1:200 with cell culture medium) was used as the negative control. Growth inhibition values for MMS were close to 100%, while the DMSO control values were close to 0% in all the test plates. Three technical replicates were performed for each biological replicate. Data was analysed using Genedata Screener software (Genedata Screener®). IC_50_ was calculated using a logarithmic approach.

### Antibacterial assays

Whole organism chemical extracts (in triplicates) were also screened against a panel of 5 different bacteria (*Escherichia coli, Klebsiella pneumoniae*, methicillin resistant *Staphylococcus aureus*, methicillin sensitive *Staphylococcus aureus* and *Mycobacterium tuberculosis*).

Gram-negative bacteria *Escherichia coli* ATCC25922 and *Klebsiella pneumoniae* ATCC700603, and Gram-positive bacteria *Staphylococcus aureus* MRSA MB5393 and MSSA ATCC29213, were used for the antibacterial MIC assays that were performed as reported by Audoin *et al*.^16^. Extracts were prepared as reported in the anticancer assays section. Briefly, a stock inoculum was seeded onto Luria-Bertani agar plates (LBA, 40 g/L) and then incubated at 37 °C overnight. Single colonies from the LB plates were inoculated on LB broth medium and incubated overnight under the same conditions with agitation. The inoculum was then diluted in order to obtain a concentration of approximately 1.1 × 10^6^ CFU/mL (MRSA and MSSA) or 5–6 × 10^5^ CFU/mL (*E. coli* and *K. pneumoniae*). For the assay, 98.4 μL/well of the diluted inoculum were mixed with 1.6 μL/well of each sample. Control antibiotics and their MIC values were: vancomycin hydrochloride (1–2 μg/mL vs MRSA and 0.5–1 μg/mL vs MSSA), aztreonam (0.0625–0.125 μg/mL vs *E. coli*), and gentamycin sulphate (4–8 μg/mL vs *K. pneumoniae*). OD was measured at 612 nm at the start (Time zero, 0 h) and at the end of the incubation in the plates (Time final, 20 h). Three technical replicates were performed for each individual assay.

Antituberculosis activity was assessed on *M. tuberculosis* H37Ra ATCC 25177 using the REMA method^17^. This method is based on fluorescence development when resazurin is reduced to resorufin by viable bacterial cells. The bacteria were grown on 96-well plates for 7 days at 5% CO_2_, 95% humidity and 37 °C, with the treatment (98.4 μL/well of inoculum and 1.6 μL/well of each sample). Maximum DMSO concentration used for the testing was 1.6%. Streptomycin sulphate with a MIC of 0.0078–0.015 μg/mL was used as control. Sample concentrations tested were 0.625, 1.25, 2.5, 5, 10, 20, 40, 80, 160 and 320 µg/mL. Fluorescence was measured after 24 hours of incubation with resazurin. Three technical replicates were performed for each individual assay. Data was analysed using Genedata Screener software (Genedata Screener®). Growth media was used as blank in all cases. Bacterial growth inhibition was calculated according to the following equation:

$$ \% Inhibition=100\ast \frac{[(TfSample-T0Sample)-(TfBlank-T0Blank)]}{[(TfGrowth-T0Growth)-(TfBlank-T0Blank)]}$$

### Semi-preparative fractionation and analysis of compounds using LC-MS

Fish bioactive extracts were fractionated by semipreparative reversed-phase HPLC-DAD (Gilson® Automated Preparative/Semipreparative HPLC systems). The extracts (diluted in DMSO) were injected onto an Agilent Zorbax SB-C8 column (9.4 mm × 250 mm, 5 µm particle size) that was eluted at a flow of 3.6 mL/min with a gradient of H_2_O:CH_3_CN (70:30 to 0:100 in 20 minutes) as eluent. Detection wavelengths used were 210 nm and 310 nm. Eight fractions were collected from each extract. Fractions were analyzed by HPLC-UV-HRMS on an Agilent 1200 RR coupled to a Bruker maXis time of flight spectrometer with electrospray ionization as described in the literature^18^.

### Statistical analysis

Statistical significance for all assays was determined by Student’s t-test using GraphPad Prim statistic software, V4.00 (GraphPad Software, San Diego, California, USA). Data were considered significant when p value was <0.05.

## Results and Discussion

### Biological activities

**Figure 1:**
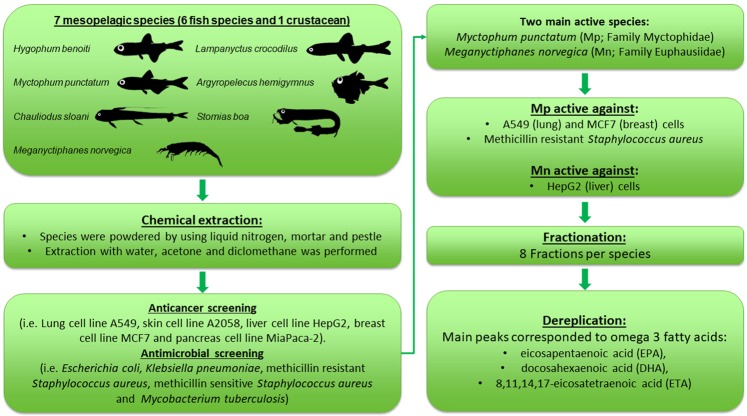
Flowchart of the experimental procedure.

Of the mesopelagic organisms screened, two species showed strong anticancer and antibacterial activities, the fish *M. punctatum* (Family Myctophidae) and the krill *M. norvegica* (Family Euphausiidae) (Fig. 1 shows the flowchart of the experimental procedure).

**Figure 2:**
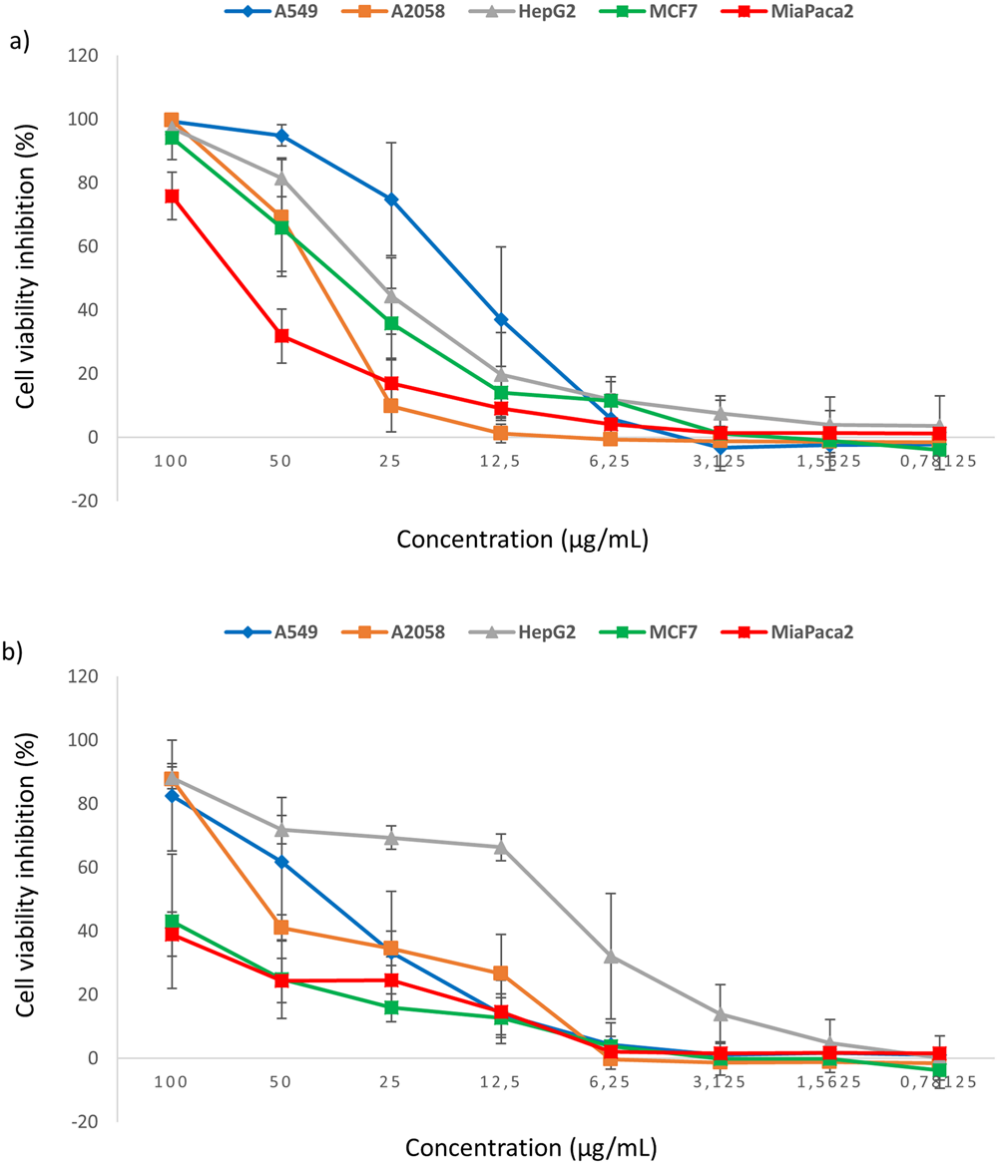
Percentage cell viability inhibition of A549 (lung), A2058 (skin), HepG2 (liver), MCF7 (breast) and MiaPaca-2 (pancreas) cancer cell lines after incubation for 72 h with 0.78125, 1.5625, 3.125, 6.25, 12.5, 25, 50, 100 µg/mL of total extract of (**a**) *M. punctatum* and (**b**) *M. norvegica*.

Regarding the anticancer assay, *M. punctatum* was more active against A549 (lung) and MCF7 (breast) cells (Fig. 2a), while *M. norvegica* was more active against HepG2 (liver) cells (Fig. 2b). Table 2 reports values of the minimum concentration inhibiting at least half cell viability (IC_50_). For *M. punctatum*, IC_50_ for A549 was 13.77–23.26 while IC_50_ for MCF7 was 25.34–29.62 μg/mL. For *M. norvegica*, IC_50_ for HepG2 cells was between 3.81 and 7.51 μg/mL.

**Table 2:** *M. punctatum* and *M. norvegica* were tested in triplicate on A549 (lung), A2058 (skin), HepG2 (liver), MCF7 (breast) and Miapaca-2 (pancreas) cancer cell lines.

Species	A549	A2058	HepG2	MCF7	MiaPaca-2
*Myctophum punctatum*	19.59 ± 5.09	41.76 ± 9.76	24.77 ± 7.51	28.12 ± 2.41	66.8 ± 3.89
*Meganyctiphanes norvegica*	42.43 ± 11.79	33.14 ± 1.66	5.34 ± 1.93	>100	>100

Each value of minimum concentration inhibiting half of cell viability (IC_50_; μg/mL) is a mean of 3 technical replicates. The lowest IC_50_ values are reported in red.

**Figure 3:**
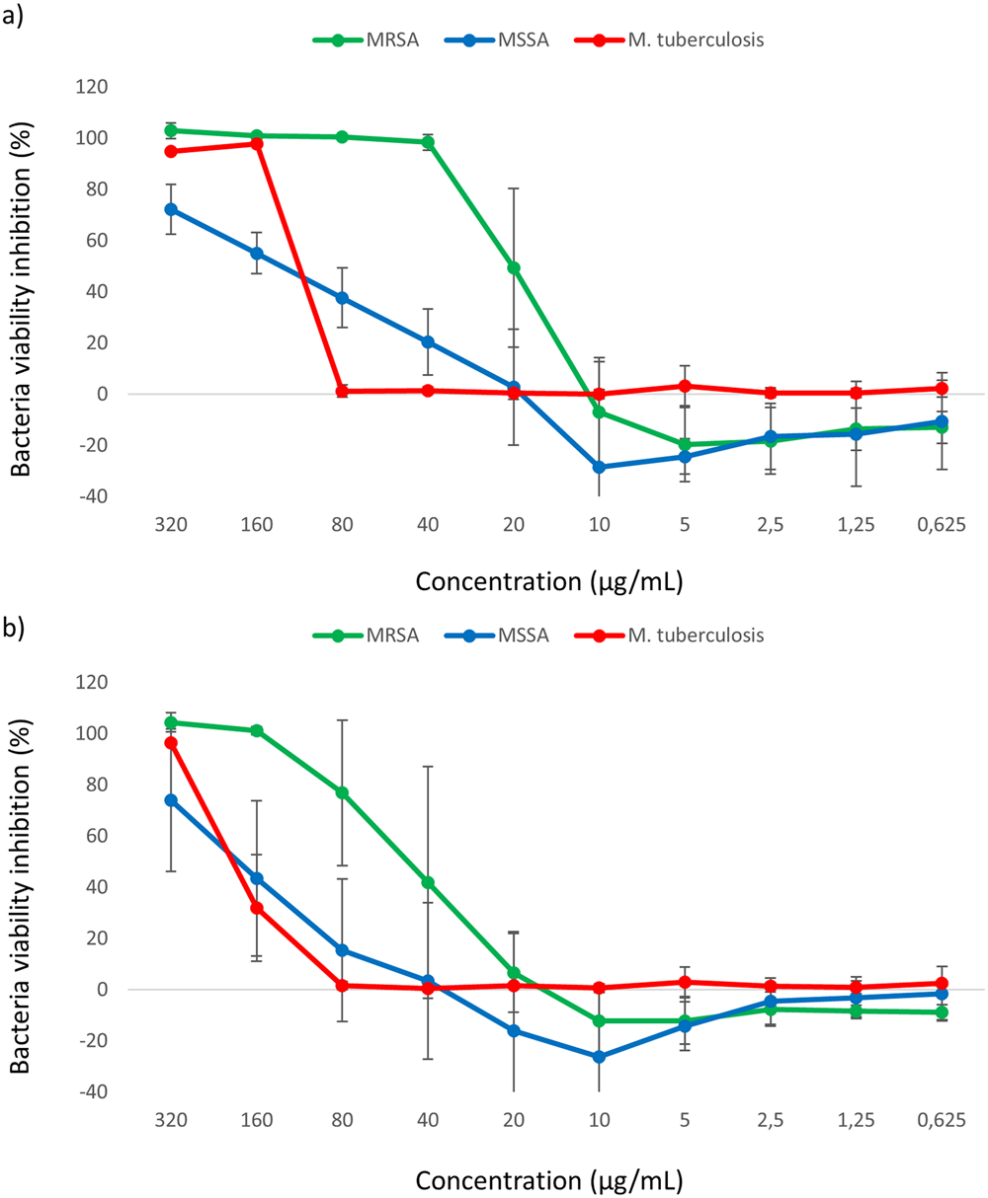
*M. punctatum* (**a**) and *M. norvegica* (**b**) extracts tested in triplicates against methicillin resistant *S. aureus* (MRSA), methicillin sensitive *S. aureus* (MSSA) and *M. tuberculosis* at different concentrations (0.625, 1.25, 2.5, 5, 10, 20, 40, 80, 160 and 320 µg/mL).

Regarding antibacterial assays, both *M. punctatum* and *M. norvegica* extracts were able to inhibit the growth of methicillin resistant *Staphylococcus aureus* (MRSA), methicillin sensitive *Staphylococcus aureus* (MSSA) and *Mycobacterium tuberculosis* depending on the tested concentrations (Fig. 3)*. M. punctatum* extract was able to inhibit 100% MRSA viability at extract concentrations between 40 and 320 µg/mL (Fig. 3a). At lower concentrations, the activity decreased. *M. punctatum* extract was also active against MSSA and *M. tuberculosis*, but only at the highest concentrations tested (320 and 160 µg/mL). *M. norvegica* extract was active against MRSA from 80–320 µg/mL, while it was active against the other bacteria only at the highest concentrations (Fig. 3b). No activity was observed against Gram-negative bacteria (i.e. *Escherichia coli* and *Klebsiella pneumoniae*).

### Dereplication results

Since isolation and characterisation of new compounds is a very time consuming and costly process^19^, dereplication by LC-UV-HRMS was performed to identify possible known compounds at an early stage using the platform available at MEDINA^20^. The anticancer and antibacterial activities were tested for 7 different mesopelagic fish species but only *M. punctatum (Mp*) and *M. norvegica* (Mn) displayed interesting anticancer and antibacterial activities. For this reason, the bioactive extracts selected for dereplication were Mp and Mn. A simple fractionation step using semipreparative HPLC-DAD was performed; 8 different fractions (F1 to F8) were obtained from each fish extract and each fraction was injected in the LC-UV-HRMS system.

**Figure 4:**
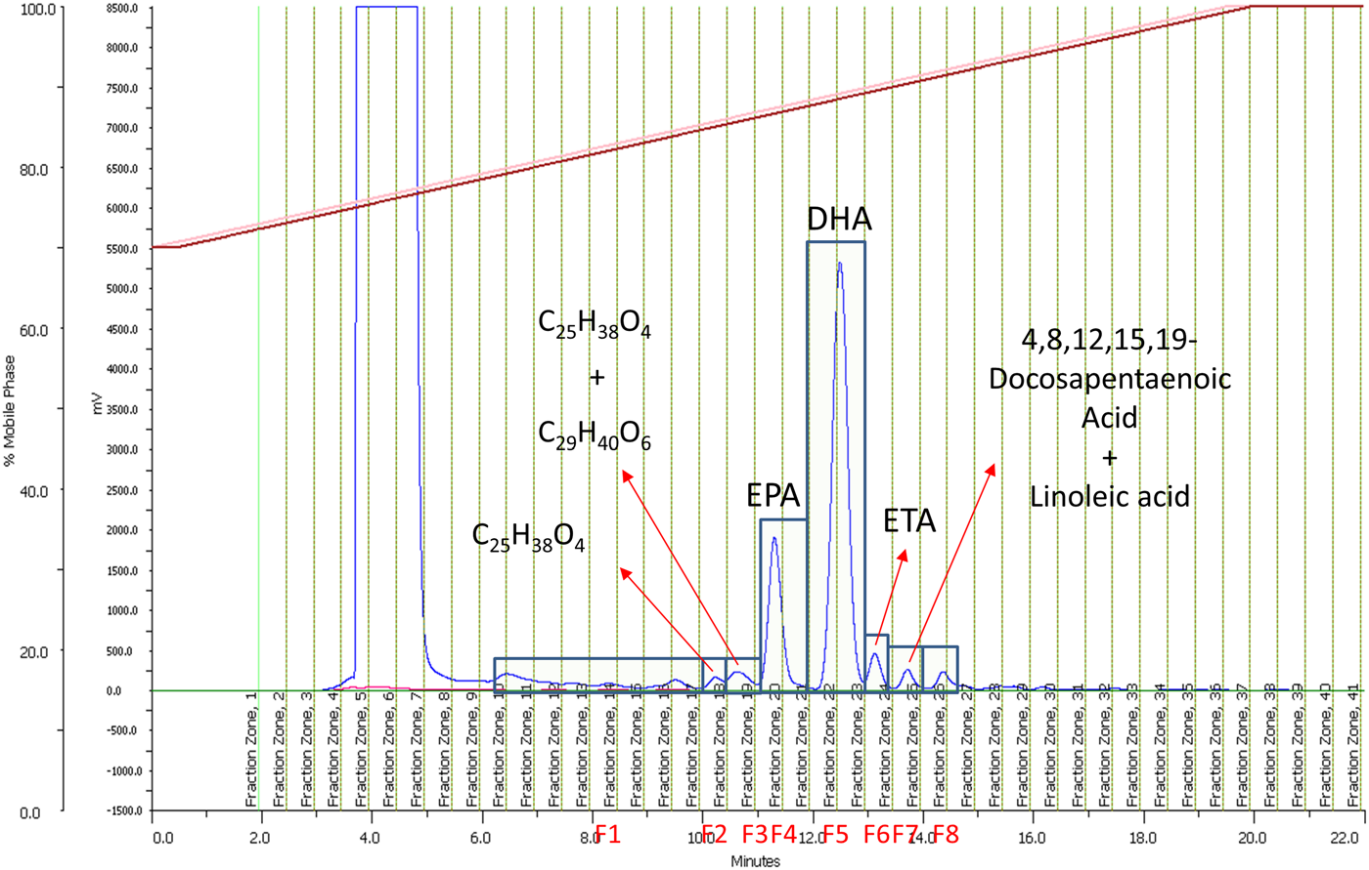
*Myctophum punctatum (Mp*) LC-UV spectrum. Fractions 1 to 8 (F1 to F8) are indicated, as well as the most abundant components detected by HPLC-TOF-HRMS.

**Figure 5:**
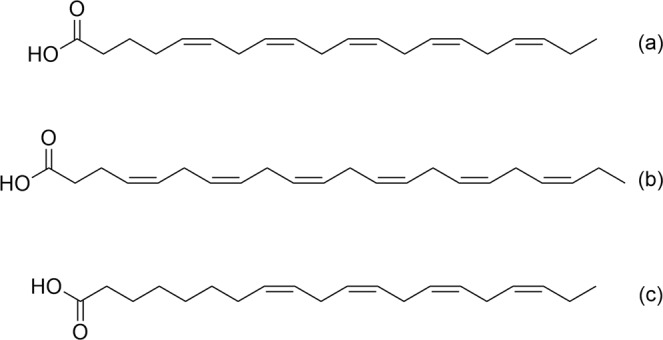
EPA (**a**), DHA (**b**) and ETA (**c**) structures.

For Mp, the most abundant components were found in fractions F4, F5 and F6 (Fig. 4). F4 had a peak with an assigned molecular formula of C_20_H_30_O_2_. Dereplication using the dictionary of natural product database^21^ (DNP) indicated that the compound had a molecular formula coincident with that of eicosapentaenoic acid (EPA, Fig. 5a). This is a known compound with an accurate mass of 302.2246, which is found in fish oil^22^. EPA, a well-known omega-3 fatty acid, has been reported to possess antibacterial activity against *Bacillus cereus* and *Staphylococcus aureus* with minimum inhibitory concentrations (MIC) of 64 µg/mL and 128 µg/mL, respectively^23^. It also displayed anticancer activity. In particular, after 72 h of incubation with EPA, lung human A549 cancer cells showed a significant reduction in cell viability^24^. EPA inhibited 50% of proliferation of A549 cells at 6.05 μM. Ogo *et al*.^25^ also showed a significant synergic effect when EPA was combined with paclitaxel or docetaxel on a human esophageal cancer cell line (TE-1), enhancing its antiproliferative effect.

F5 was a peak corresponding to the formula C_22_H_32_O_2_. Dereplication using the DNP database indicated that the compound had the same molecular formula as docosahexaenoic acid (DHA, Fig. 5b), an omega-3 fatty acid with an accurate mass of 328.2402. DHA is found in fish oil from several species as widely reported in the literature^22^. DHA has been reported to be active against breast (MDA-MB-231, MCF-7), pancreatic (MiaPaca-2) and colorectal (CaCo-2, SW-620) cancer cell lines at a range of concentrations between 10 and 100 µM^26^.

A molecular formula of C_20_H_32_O_2_ was assigned to F6, which corresponds to the compound 8,11,14,17-eicosatetraenoic acid (ETA, Fig. 5c). This is a known compound closely related to EPA (one less double bond at position 5) with an accurate mass of 304.2402. ETA is an ω-3 fatty acid naturally present in fish oils at levels of around 1–2%^27^. No biological activities have been reported for this compound.

F2 and F3 also contained some minor components with molecular formulae C_25_H_38_O_4_ (acc. mass 402.2765) and C_29_H_40_O_6_ (acc. mass 484.2817), most likely corresponding to polyunsaturated fatty acids (PUFAs) due to their retention time and formulae. F7 contained traces of linoleic acid (C_18_H_32_O_2_, acc. mass 280.2402) and 4,8,12,15,19-docosapentaenoic acid (C_22_H_34_O_2_, acc. mass 330.2556). Linoleic acid is an ω-6 fatty acid present in various fish species^28^ whose anticancer activity has been studied^29,30^. However, previous studies did not conclude whether or not linoleic acid is active against the cancer cells tested (including colorectal, breast and lung cancer cell lines). 4,8,12,15,19-Docosapentaenoic acid is an ω-3 fatty acid with an unusual distribution of its double bonds. It was isolated for the first time in the Japanese sardine *Clupanodon melanostica*^31^. The biological activity of this fatty acid is not reported in the literature. F1 and F8 did not show the presence of any significant components when analysed.

**Figure 6:**
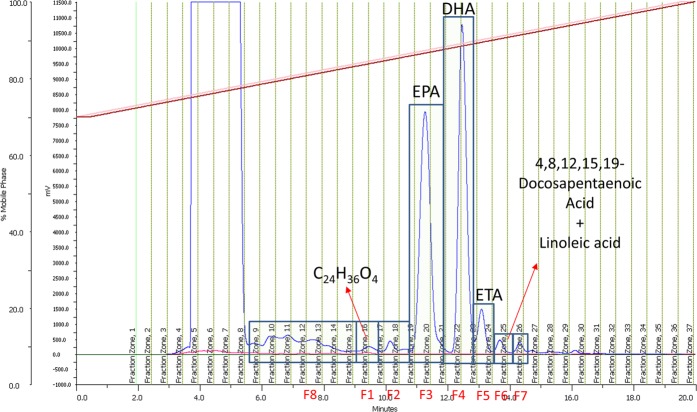
*M. norvegica* (Mn) LC-UV spectrum. Fractions 1 to 8 (F1 to F8) are indicated, as well as the components detected by HPLC-TOF-HRMS.

For Mn, the most abundant components were found in fractions 3 and 4 (Fig. 6). F3 corresponded to a peak assigned to the molecular formula C_20_H_30_O_2_, identical to the fraction F4 of the sample Mp and, hence, EPA. The main component of F4 was assigned the molecular formula C_22_H_32_O_2_, and identified as DHA, the same major component that was also present in F5 of Mp extract. F1 also contained a minor peak with a molecular formula of C_24_H_36_O_4_ (acc. mass 402.2765), most likely to be a PUFA due to its retention time and formula. F5 and F6 contained traces of linoleic acid (C_18_H_32_O_2_, acc. mass 280.2402), 4,8,12,15,19-docosapentaenoic acid (C_22_H_34_O_2_, acc. mass 330.2556) and 8,11,14,17-eicosatetraenoic acid (C_20_H_32_O_2_, acc. mass 304.2402). F2, F7 and F8 did not show any significant components by LC/MS.

Even if similar, also the combination of the various components present in the extracts of the two species may be responsible for the biological activities observed in this study. Forthcoming studies will concern the sampling of more individuals of the two active species and further chemical fractionation in order to isolate and screen each extract component. Some of the detected compounds have already displayed anticancer and/or antibacterial activity in studies reported in the literature^23–25^. Major compounds such as EPA, DHA and ETA are especially interesting for the food and supplement industries^32^. This is the first time that such activities have been found for these mesopelagic species and we here propose them as new abundant sources of compounds useful for human health.
